# Supplementary material for: Knowledge and Attitudes of Cannabidiol in Croatia among Students, Physicians, and Pharmacists
Source: Pharmacy (Basel). 2023 Dec 23;12(1):2. doi: 10.3390/pharmacy12010002 (PMC10801513; doi:10.3390/pharmacy12010002)
Supplement: Supplementary file 1 [file pharmacy-12-00002-s001.zip › Attitudes and knowledge about the use of medical CBD/Attitudes and knowledge of doctors and pharmacists about the use of medical CBD.pdf]

Stavovi i znanja liječnika i ljekarnika o upotrebi medicinskog CBD-a

Poštovani ispitanici, ova anketa je u potpunosti anonimna i provodi se u svrhu znanstvenog istraživanja o stavovima i znanjima o upotrebi medicinskog CBD-a.
Posljednjih godina se provodi sve više istraživanja vezanih za upotrebu i dobrobit CBD-a.
Na dostupnim medicinskim bazama se gotovo godišnje objavi na stotine znanstvenih istraživanja. Na stranici: https://pubmed.ncbi.nlm.nih.gov/?term=medical+CBD&filter=datesearch.y_1 u kojoj se "medical CBD" koristila kao ključna riječ, uz filter "publication date" od 1 godine, dobiveni su podatci o 650 naslova.
To dovoljno govori o važnosti informacija o znanju liječnika i ljekarnika, njihovoj pravovremenoj edukaciji, a sve kako bi se u budućnosti snašli i uspješno donosili važne odluke u eventualnom korištenju medicinskog CBD-a.
Istraživači koji sudjeluju u ovom istraživanju su nastavnici Sveučilišta u Splitu, Zagrebu i Osijeku.
Nakon završene i obrađene ankete, rezultati će se usporediti s rezultatima sličnih anketa susjednih država.
Molimo Vas da anketu ispunite, a nadamo se da Vam nećemo oduzeti puno vremena. Još jednom hvala na sudjelovanju.

Istraživači

* Indicates required question

1. 1. Spol? *

Mark only one oval.

- Ž
- M

2. 2. Odaberite pripadajuće zanimanje *

Mark only one oval.

- Liječnik opće prakse
- Liječnik specijalist
- Ljekarnik
- Ljekarnik specijalist

3. 3. Navedite koju specijalizaciju posjedujete (za ispitanike sa specijalizacijom)?

____

4. 4. Koliko dugo godina radite u praksi? *

Mark only one oval.

- 1-5
- 6-10
- 10-20
- 20-30
- 30-40
- više od 40

5. 5. Imate li saznanja o CBD-u? *

Mark only one oval.

- da
- ne

6. 6. Jeste li ikada konzumirali CBD? *

Mark only one oval.

- da
- ne

7. 7. Mislite li da je CBD štetan za zdravlje? *

Mark only one oval.

	1	2	3	4	5	
u po						u potpunosti se slažem

8. 8. Mislite li da je primjena CBD-a u liječenju učinkovita? *

Mark only one oval.

	1	2	3	4	5	
u po						u potpunosti se slažem

9. 9. Kroz svoje formalno obrazovanje imao/imala sam edukaciju o korištenju CBD-a u medicinske svrhe. *

Mark only one oval.

- da
- ne

10. 10. Smatram da mi treba dodatna edukacija o korištenju CBD-a u medicinske svrhe. *

Mark only one oval.

	1	2	3	4	5	
u po	◯	◯	◯	◯	◯	u potpunosti se slažem

11. 11. Upoznat/a sam s rizicima korištenja CBD-a. *

Mark only one oval.

	1	2	3	4	5	
u po	◯	◯	◯	◯	◯	u potpunosti se slažem

12. 12. Upoznat/a sam s dobrobitima korištenja CBD-a. *

Mark only one oval.

	1	2	3	4	5	
u po	◯	◯	◯	◯	◯	u potpunosti se slažem

13. 13. Korištenje CBD-a ima pozitivne učinke na tjelesno zdravlje. *

Mark only one oval.

	1	2	3	4	5	
u po	◯	◯	◯	◯	◯	u potpunosti se slažem

14. 14. Korištenje CBD-a ima pozitivne učinke na mentalno zdravlje. *

Mark only one oval.

	1	2	3	4	5	
u po	◯	◯	◯	◯	◯	u potpunosti se slažem

15. 15. CBD pomaže pacijentima s kronično iscrpljujućim stanjima. *

Mark only one oval.

	1	2	3	4	5	
u po	◯	◯	◯	◯	◯	u potpunosti se slažem

16. 16. Korištenje CBD-a izaziva tjelesnu ovisnost. *

Mark only one oval.

	1	2	3	4	5	
u po	◯	◯	◯	◯	◯	u potpunosti se slažem

17. 17. Korištenje CBD-a izaziva psihičku ovisnost. *

Mark only one oval.

	1	2	3	4	5	
u po	◯	◯	◯	◯	◯	u potpunosti se slažem

18. 18. Korištenje CBD-a može dovesti do ovisnosti o drugim opiodima i drogama. *

Mark only one oval.

	1	2	3	4	5	
u po	◯	◯	◯	◯	◯	u potpunosti se slažem

19. 19. CBD izaziva osjećaj euforije. *

Mark only one oval.

	1	2	3	4	5	
u po	◯	◯	◯	◯	◯	u potpunosti se slažem

20. 20. Prema Vašim saznanjima što mislite za koje indikacije je FDA (Food and drug administration) odobrila lijekove s CBD-om? *

Check all that apply.

- ☐ Mučnina povezana s kemoterapijom
- ☐ Kronična neuropatska bol
- ☐ EPI napadi kod Lennox- Gastaut i Dravet sindroma
- ☐ Deprsivni poremećaji
- ☐ Parkinsonova bolest
- ☐ Tuberozna skleroza
- ☐ Bolovi kod malignih oboljenja

21. 21. Prema Vašim saznanjima koje neželjene učinke uzrokuje CBD? *

Check all that apply.

- [ ] Anemija
- [ ] Tahikardija
- [ ] Proljev i povraćanje
- [ ] Glaukom
- [ ] Smanjen tek
- [ ] Hiperglikemija
- [ ] Somnolencija

22. 22. Prema Vašim saznanjima koji lijekovi imaju umjerene ili velike interakcije s CBD-om? *

Check all that apply.

- [ ] Paracetamol
- [ ] Valproat
- [ ] Omeprazol
- [ ] Karbamazepin
- [ ] Ibuprofen
- [ ] Rifampicin
- [ ] Amoksicilin
- [ ] Everolimus
- [ ] Klobazam
- [ ] Fenhidramin

23. 23. Prema Vašim saznanjima kod kojih stanja je potreban oprez pri upotrebi CBD-a? *

Check all that apply.

- [ ] Srčana aritmija
- [ ] Hepatocelularno oštećenje
- [ ] Glaukom
- [ ] Somnolencija
- [ ] Karcinom
- [ ] Smanjena tjelesna težina
- [ ] Trudnoća
- [ ] Suicidalno ponašanje
- [ ] Somnolencija i sedacija

24. 24. Podupirem korištenje CBD-a kod: *

Check all that apply.

- [ ] Palijativnih bolesnika
- [ ] Ublažavanje boli kod oboljelih od karcinoma
- [ ] Nuspojava kemoterapije
- [ ] Multiple skleroze
- [ ] Neuropatske boli
- [ ] Kronične boli
- [ ] PTSP
- [ ] Nesanice
- [ ] Kronove bolesti
- [ ] Glaukoma
- [ ] Hepatitisa C
- [ ] Mišične spastičnosti
- [ ] HIV-a
- [ ] Traumatske ozljede mozga
- [ ] ALS-a
- [ ] Alzheimerove bolesti
- [ ] Anoreksije
- [ ] Parkinsonove bolesti
- [ ] Migrene

25. 25. Smatram kako bi preporučivanje/prepisivanje CBD-a moglo smanjiti upotrebu opioida kod kronične boli. *

Mark only one oval.

- DA
- NE

26. 26. Smatram da imam dovoljno znanja o korištenju CBD-a u medicinske svrhe te da ga mogu preporučiti pacijentima. *

Mark only one oval.

- DA
- NE

27. 27. Jeste li do sada u svojoj praksi pacijentima preporučili/prepisali korištenje CBD-a? *

Mark only one oval.

- NE
- Da, samo jednom
- Da, više puta
- Da, često pacijentima specifičnih dijagnoza

28. 28. Smatram da bi zdravstveno osiguranje trebalo pokrivati troškove CBD-a ukoliko ga liječnik prepiše kao terapiju. *

Mark only one oval.

- DA
- NE

29. 29. Jeste li ikada pročitali neki znanstveno-stručni rad o CBD-u?

Mark only one oval.

- DA
- Ne

30. 30. Obrazovni kurikulumi liječnika, zdravstvenih djelatnika i farmaceuta trebali bi sadržavati predmete o upotrebi CBD-a u medicinske svrhe. *

Mark only one oval.

	1	2	3	4	5	
u po						u potpunosti se slažem

31. 31. U kojoj županiji živite? *

Mark only one oval.

- Bjelovarsko-bilogorska
- Brodsko-posavska
- Dubovačko-neretvanska
- Istarska
- Karlovačka
- Koprivničko-križevačka
- Krapinsko-zagorska
- Ličko-senjska
- Međimurska
- Osječko-baranjska
- Požeško-slavonska
- Primorsko-goranska
- Sisačko-moslavačka
- Splitsko-dalmatinska
- Šibensko-kninska
- Varaždinska
- Virovitičko-podravska
- Vukovarsko-srijemska
- Zadarska
- Zagrebačka
- Grad Zagreb

Anketa

Zahvaljujemo Vam na sudjelovanju u ovoj anketi. Svojim odgovorima doprinijeli ste boljem razumijevanju trenutnog stanja o
znanjima i stavovima upotrebe medicinskog CBD-a te potrebom za eventualnm dodatnim edukacijama.
Istraživači

This content is neither created nor endorsed by Google.

Google Forms
