# Supplementary material for: Knowledge and Attitudes of Cannabidiol in Croatia among Students, Physicians, and Pharmacists
Source: Pharmacy (Basel). 2023 Dec 23;12(1):2. doi: 10.3390/pharmacy12010002 (PMC10801513; doi:10.3390/pharmacy12010002)
Supplement: Supplementary file 1 [file pharmacy-12-00002-s001.zip › Attitudes and knowledge about the use of medical CBD/Attitudes and knowledge of students about the use of medical CBD.pdf]

Stavovi i znanja studenata o upotrebi medicinskog CBD-a

Poštovani ispitanici, ova anketa je u potpunosti anonimna i provodi se u svrhu znanstvenog istraživanja o stavovima i znanjima o upotrebi medicinskog CBD-a.
Posljednjih godina se provodi sve više istraživanja vezanih za upotrebu i dobrobit CBD-a. Na dostupnim medicinskim bazama se gotovo godišnje objavi na stotine znanstvenih istraživanja. Na stranici: https://pubmed.ncbi.nlm.nih.gov/?term=medical+CBD&filter=datesearch.y_1 u kojoj se "medical CBD" koristila kao ključna riječ, uz filter "publication date" od 1 godine, dobiveni su podatci o 650 naslova.
To dovoljno govori o važnosti informacija o znanju studenata zdravstvenih struka, njihovoj pravovremenoj edukaciji, a sve kako bi se u budućnosti snašli i uspješno donosili važne odluke u eventualnom korištenju medicinskog CBD-a.
Istraživači koji sudjeluju u ovom istraživanju su nastavnici Sveučilišta u Splitu, Zagrebu i Osijeku.
Nakon završene i obrađene ankete, rezultati će se usporediti s rezultatima sličnih anketa susjednih država.
Molimo Vas da anketu ispunite, a nadamo se da Vam nećemo oduzeti puno vremena. Još jednom hvala na sudjelovanju.

Istraživači

* Indicates required question

1. 1. Spol? *

Mark only one oval.

- ( ) Ž
- ( ) M

2. 2. Koji studijski program- fakultet pohađate? *

Mark only one oval.

- Studij medicine - Medicinski fakultet Split
- Studij farmacije - Medicinski fakultet Split
- Farmaceutsko-biokemijski fakultet Sveučilišta u Zagrebu
- Medicinski fakultet Sveučilišta u Osijeku
- Sveučilišni odjel zdravstvenih studija Sveučilišta u Splitu (preddiplomski studiji)
- Sveučilišni odjel zdravstvenih studija Sveučilišta u Splitu (diplomski studiji)

3. 3. Godina studija? *

Mark only one oval.

- 1
- 2
- 3
- 4
- 5
- 6

4. 4. Imate li saznanja o CBD-u? *

Mark only one oval.

- da
- ne

5. 5. Jeste li ikada konzumirali CBD? *

Mark only one oval.

- da
- ne

6. 6. Mislite li da je CBD štetan za zdravlje? *

Mark only one oval.

	1	2	3	4	5	
u po						u potpunosti se slažem

7. 7. Mislite li da je primjena CBD-a u liječenju učinkovita? *

Mark only one oval.

	1	2	3	4	5	
u po						u potpunosti se slažem

8. 8. Kroz svoje formalno obrazovanje imao/imala sam edukaciju o korištenju CBD-a u medicinske svrhe. *

Mark only one oval.

- da
- ne

9. 9. Smatram da mi treba dodatna edukacija o korištenju CBD-a u medicinske svrhe. *

Mark only one oval.

	1	2	3	4	5	
u po	◯	◯	◯	◯	◯	u potpunosti se slažem

10. 10. Upoznat/a sam s rizicima korištenja CBD-a. *

Mark only one oval.

	1	2	3	4	5	
u po	◯	◯	◯	◯	◯	u potpunosti se slažem

11. 11. Upoznat/a sam s dobrobitima korištenja CBD-a. *

Mark only one oval.

	1	2	3	4	5	
u po	◯	◯	◯	◯	◯	u potpunosti se slažem

12. 12. Korištenje CBD-a ima pozitivne učinke na tjelesno zdravlje. *

Mark only one oval.

	1	2	3	4	5	
u po	◯	◯	◯	◯	◯	u potpunosti se slažem

13. 13. Korištenje CBD-a ima pozitivne učinke na mentalno zdravlje. *

Mark only one oval.

	1	2	3	4	5	
u po	◯	◯	◯	◯	◯	u potpunosti se slažem

14. 14. CBD pomaže pacijentima s kronično iscrpljujućim stanjima. *

Mark only one oval.

	1	2	3	4	5	
u po	◯	◯	◯	◯	◯	u potpunosti se slažem

15. 15. Korištenje CBD-a izaziva tjelesnu ovisnost. *

Mark only one oval.

	1	2	3	4	5	
u po	◯	◯	◯	◯	◯	u potpunosti se slažem

16. 16. Korištenje CBD-a izaziva psihičku ovisnost. *

Mark only one oval.

	1	2	3	4	5	
u po	◯	◯	◯	◯	◯	u potpunosti se slažem

17. 17. Korištenje CBD-a može dovesti do ovisnosti o drugim opiodima i drogama. *

Mark only one oval.

	1	2	3	4	5	
u po	◯	◯	◯	◯	◯	u potpunosti se slažem

18. 18. CBD izaziva osjećaj euforije. *

Mark only one oval.

	1	2	3	4	5	
u po	◯	◯	◯	◯	◯	u potpunosti se slažem

19. 19. Jeste li ikada pročitali neki znanstveno-stručni rad o CBD-u? *

Mark only one oval.

◯ DA

◯ Ne

20. 20. Obrazovni kurikulumi liječnika, zdravstvenih djelatnika i farmaceuta trebali bi sadržavati predmete o upotrebi CBD-a u medicinske svrhe. *

Mark only one oval.

	1	2	3	4	5	
u po	◯	◯	◯	◯	◯	u potpunosti se slažem

Anketa

Zahvaljujemo Vam na sudjelovanju u ovoj anketi. Svojim odgovorima doprinijeli ste boljem razumijevanju trenutnog stanja o
znanjima i stavovima upotrebe medicinskog CBD-a te potrebom za eventualnm dodatnim edukacijama.
Istraživači

This content is neither created nor endorsed by Google.

Google Forms
